# Gamma Delta T-Cell Receptor Lymphoma Causing Bilateral Pulmonary Embolism

**DOI:** 10.7759/cureus.18072

**Published:** 2021-09-18

**Authors:** Kanksha Peddi, Brandon Wiggins, Omar Choudhury, Mark Ortolani

**Affiliations:** 1 Internal Medicine, Ascension Genesys Hospital, Grand Blanc, USA; 2 Neurology, Henry Ford Health System, Detroit, USA; 3 Emergency Medicine, Ascension Genesys Hospital, Grand Blanc, USA

**Keywords:** gamma delta t-cell, cutaneous t cell lymphoma, pulmonary embolism (pe), t-cell lymphoma, acute pulmonary embolism, hypercoagulable, venous thromboembolism (vte)

## Abstract

Pulmonary embolism is a previously uncharacterized complication of primary cutaneous gamma delta T-cell lymphoma (PCGDTL), a type of cutaneous T-cell receptor lymphoma that accounts for less than 1% of non-Hodgkin’s lymphomas. We report the first documented case of bilateral pulmonary embolism in the setting of PCGDTL in a 30-year-old woman who presented with acute dyspnea.

## Introduction

Primary cutaneous gamma delta T-cell lymphoma (PCGDTL) is a rare cutaneous form of lymphoma. It is an aggressive subset of natural killer T cell (TN/K) or natural killer cell lymphomas. TN/K lymphomas make up approximately 6% of all non-Hodgkin’s lymphomas and PCGDTL comprises less than 1% of this group [1,2]. With only a handful of documented cases in the literature, and no documented cases complicated by pulmonary embolism, the clinical manifestations require further characterization. Prognosis is poor with a median survival time of approximately 18 months [1]. The clinical presentation involves cutaneous manifestations such as papules or plaques and nodules with ulcerations. Underlying panniculitis with overlying epidermal necrosis is a feature reported with most lesions that present on the extremities for these patients. It has also commonly been reported to involve the trunk and face.

Symptoms of PCGDTL are often typical "B symptoms" such as fever, chills, night sweats, fatigue, and weight loss with the presence of aforementioned cutaneous lesions, which are typically painful. Abdominal, chest pain, and particularly bone pain have been reported but have not yet been investigated further. Prior to this case report, there has yet to be any reported case of pulmonary embolism as a complication of PCGDTL.

## Case presentation

A 30-year-old female with a past medical history of PCGDTL presented for sudden onset dyspnea. The patient reported sudden onset of dyspnea with activity and at rest. She described her pain as pleuritic associated with bilateral shoulder pain. She reported positional exacerbation of her dyspnea. She denied any nausea, vomiting, fevers, hemoptysis, fevers, chills, cough, abdominal pain, changes in bowel or urinary habits, or other complaints at the time. The patient denied any history of blood clots, history of oral contraceptives, or other hormone use. On arrival to the emergency department, the patient was tachycardic, afebrile, normotensive, and saturating 100% on 4 L nasal cannula. Physical examination was significant for an alert and awake but distressed appearing female, with lungs that were clear to auscultation bilaterally with no wheezing, stridor.

**Figure 1:**
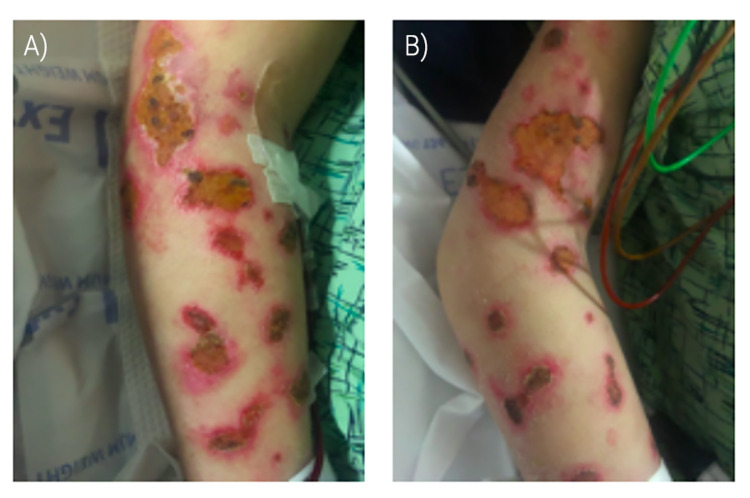
Cutaneous manifestations of PCGDTL (A) Left upper extremity with diffuse patchy hypopigmented lesions, interspersed, oval or discoid, excoriated and ulcerated with occasional scabbing, without drainage or purulence. (B) Similar skin findings of the right upper extremity. PCGDTL: primary cutaneous gamma delta T-cell lymphoma

Two months prior to presentation, the patient presented with febrile neutropenia and sepsis and was diagnosed with T-cell lymphoma. Initial therapy involved antibiotics and supportive care with acetaminophen and fluid resuscitation which led to clinical improvement. Skin examination during that encounter was significant for several lesions in the bilateral arms and legs and back. The lesions were flat, erythematous with crusting consistent with gamma delta lymphoma with some areas of foul-smelling discharge. Upper extremities were notable for patchy hypopigmented lesions that were independent, interspersed, oval or discoid, excoriated, and ulcerated with occasional scabbing, without drainage or purulence. On the lower extremities, there were diffuse interspersed patchy hypopigmented lesions also oval or discoid in shape with ulceration and excoriation. However, these lesions demonstrated prominent scabbing and mild drainage with malodorous purulence. Additionally, all of the lower extremity lesions had moderate surrounding erythema (Figure 1). The patient’s skin lesions showed remarkable improvement with chemotherapy. In this encounter, the patient does not endorse any further integumentary symptoms or concerns.

The patient has a past medical history of primary cutaneous gamma delta T-cell lymphoma on chemotherapy, bi-cytopenia, and Takotsubo cardiomyopathy. Investigations include the below findings on the initial presentation (Table 1). 

**Table 1:** Initial laboratory findings

Laboratory Values	Measured	Normal Range
White Blood Cell Count (WBC)	2.0 k/uL	4.5-11.0 k/uL
Hemoglobin (Hb)	10.7 g/dL	11.0-16.2 g/dL
Hematocrit (Hct)	31.1%	36-46%
Mean Corpuscular Volume (MCV)	91.0 Fl	80.0-100.0 Fl
Absolute Neutrophil Count (ANC)	1.4 k/uL	1.0-8.0 k/uL
Sodium (Na)	138 mmol/L	136-144 mmol/L
Potassium (K+)	3.3 mmol/L	3.6-5.1 mmol/L
Bicarbonate (HCO3-)	24 mmol/L	20-30 mmol/L
Blood Urea Nitrogen (BUN)	13 mg/dL	8-26 mg/dL
Troponin	0.02 ng/mL	0.00-0.03 ng/mL
N-Terminal Pro-B-Type Natriuretic Peptide	<10 pg/mL	10-100 pg/mL
Lactic Acid	1.50 mmol/L	0.50-2.20 mmol/L

**Figure 2:**
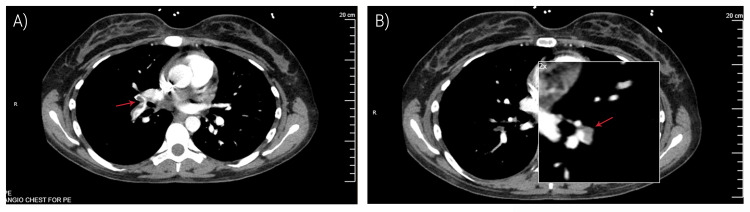
Bilateral pulmonary embolism on CT angiography (A) Filling defect within the interlobar artery on the right extending to the right middle and lower lobe segmental pulmonary arteries. (B) Subsegmental pulmonary arteries in the left lower lobe also demonstrated filling defects.

Differential diagnoses included pulmonary embolism, myocardial infarction, pneumonia, and drug-induced vasospasm. Diagnostic imaging obtained revealed a chest X-ray that was unremarkable. The patient underwent a lower extremity venous doppler ultrasound of the lower extremities which was negative for deep venous thrombosis (DVT). CT angiography (CTA) of the chest demonstrated a filling defect within the interlobar artery on the right extending to the right middle and lower lobe segmental pulmonary arteries. Subsegmental pulmonary arteries in the left lower lobe also demonstrated filling defects (Figure 2). This was consistent with bilateral pulmonary emboli without evidence of right heart strain. A small pericardial effusion was also visualized with no pleural effusions. Additionally, patchy peripheral opacities in the right lower lobe were seen, thought to be secondary to the development of an infiltrate.

Upon confirmation of pulmonary embolisms on CTA, the patient was started on high-intensity heparin (HIH) infusion for pulmonary embolism (PE) and given intravenous (IV) vancomycin for pulmonary infiltrate suspicious for pneumonia. Vancomycin was subsequently switched to ceftriaxone as the patient developed anaphylactoid red man syndrome (RMS) for which IV diphenhydramine was administered. Cardiology and hematology were consulted. Hemodynamic stability precluded the need for thrombectomy despite admission to the intensive care unit (ICU). Approximately three days after her initial presentation, hypoxemia resolved, tachycardia improved, and she transitioned from heparin infusion to oral anticoagulation. At discharge, she was hemodynamically stable and prescribed a 10-day course of cefuroxime and apixaban for six months.

## Discussion

Primary cutaneous T-cell lymphomas (PCTCL) describe T-cell lymphomas that are specifically present in the skin without evidence of extracutaneous extension [1]. PCTCL is further divided into four rare subtypes including primary cutaneous gamma delta T-cell lymphoma, primary cutaneous aggressive epidermotropic cluster of differentiation (CD)-8 cytotoxic T-cell lymphoma, primary cutaneous acral CD8 T-cell, and primary cutaneous CD4 small/medium T-cell lymphoproliferative disorder [2]. Gamma delta T-cell lymphoma is defined as a T-cell lymphoma consisting of activated gamma delta T-cells with cytotoxic phenotype and rapidly progressive nature [1]. Gamma delta T-cell lymphoma is very rare and consists of less than 1% of cutaneous T-cell lymphomas [1]. Gamma delta T-cell lymphoma has been reported to affect patients between the ages of 20 years and 90 years but is typically seen in middle-aged patients or the elderly with a mean age of 60 years [3]. 

Gamma delta T cells are antigen-presenting cells that are so disordered that persistent antigenic stimulation is thought to be the cause of this specific and rare type of lymphoma [4]. Gamma delta T-cell lymphoma usually occurs on the extremities, can involve subcutaneous, dermal or epidermal layers, and can appear as plaques, nodules, or frank tumors [5]. Gamma delta T-cell lymphoma is identified by being uniquely positive for CD2, CD3, CD56, while being negative for CD4, CD5, CD8, beta F1, and Epstein-Barr virus (EBV) [6]. Gamma delta T cell can be diagnosed via incisional or punch biopsy. Recently, T-cell receptor antibody has gained utility in helping to differentiate delta gamma T-cell lymphoma from other cutaneous lymphomas [7]. 

Venous thromboembolism, including pulmonary embolisms, is fairly common in people with malignancies due to the hypercoagulable nature of the disease process, with incidence of up to 15% [8]. Venous thromboembolism typically occurs as a result of one or multiple factors including venous stasis, endothelial damage, and/or alterations of blood products [9]. In malignancy, the hypercoagulable state is usually the reason for venous thromboembolism (VTE), owing to alterations in the make-up of blood, including increased amount of tissue factors, platelet activators, and other cancer pro-coagulants [10]. Certain types of cancers are vastly more likely to cause venous thromboembolism, including lung cancer (17%), pancreatic cancer (10%), colorectal cancer (8%), kidney cancer (8%), and prostate cancer (7%) being the most common [9]. In malignancy, a hypercoagulable state is usually the reason for VTE, owing to alterations in the make-up of blood, including an increased amount of tissue factors, platelet activators, and other cancer pro-coagulants [10]. Upon further investigation, according to PubMed inquiries, it appears this is the first reported case of pulmonary embolism in delta gamma T-cell lymphoma, leading to a very important revelation about the potential for this rare disease to have significant and unexpected complications like pulmonary embolism [11].

## Conclusions

Though B symptoms and cutaneous manifestations have been described in the literature, a case of PE as a complication of PCGDTL has yet to be characterized. This report of a 30-year old woman presenting with acute dyspnea characterizes one such case. Though VTE including PE is a common manifestation of malignancy, it occurs more frequently in solid tumor malignancies as opposed to liquid tumor malignancies, especially the type of non-Hodgkin lymphoma aforementioned. Though rare, it is important to characterize new complications of PCGDTL, and particularly those that may be fatal or manageable during a hospital stay or possibly prevented with prophylaxis.
